# Active transfer learning for audiogram estimation

**DOI:** 10.3389/fdgth.2024.1267799

**Published:** 2024-03-11

**Authors:** Hossana Twinomurinzi, Herman Myburgh, Dennis L. Barbour

**Affiliations:** ^1^ Department of Electrical, Electronic and Computer Engineering, University of Pretoria, Pretoria, South Africa; ^2^ Department of Biomedical Engineering, Washington University in St. Louis, St. Louis, MO, United States

**Keywords:** active learning, active transfer learning, audiogram estimation, audiology, audiometry, transfer learning

## Abstract

Computational audiology (CA) has grown over the last few years with the improvement of computing power and the growth of machine learning (ML) models. There are today several audiogram databases which have been used to improve the accuracy of CA models as well as reduce testing time and diagnostic complexity. However, these CA models have mainly been trained on single populations. This study integrated contextual and prior knowledge from audiogram databases of multiple populations as informative priors to estimate audiograms more precisely using two mechanisms: (1) a mapping function drawn from feature-based homogeneous Transfer Learning (TL) also known as Domain Adaptation (DA) and (2) Active Learning (Uncertainty Sampling) using a stream-based query mechanism. Simulations of the Active Transfer Learning (ATL) model were tested against a traditional adaptive staircase method akin to the Hughson-Westlake (HW) method for the left ear at frequencies ω=0.25,0.5,1,2,4,8 kHz, resulting in accuracy and reliability improvements. ATL improved HW tests from a mean of 41.3 sound stimuli presentations and reliability of ±9.02 dB down to 25.3±1.04 dB. Integrating multiple databases also resulted in classifying the audiograms into 18 phenotypes, which means that with increasing data-driven CA, higher precision is achievable, and a possible re-conceptualisation of the notion of phenotype classifications might be required. The study contributes to CA in identifying an ATL mechanism to leverage existing audiogram databases and CA models across different population groups. Further studies can be done for other psychophysical phenomena using ATL.

## 1 Introduction

The World Health Organisation (WHO) estimates that more than 5% of the world’s population, approximately 430 million people, currently have a degree of hearing loss and require rehabilitation (1). The WHO further estimates that by 2,050, 2.5 billion people will have a degree of hearing loss with 700 million requiring rehabilitation. While the challenge of hearing loss is more common in low- to middle-income populations, the traditional assessment of hearing is not easily accessible to these populations because it is time-consuming and expensive.

Computational audiology (CA) has been shown to make hearing assessment more accessible (2) by for example, reducing diagnostic time from hours to a few minutes with significantly fewer stimuli presentations (3, 4), assessing both ears simultaneously (5), making distinctions at finer frequencies and intensity levels (6, 7) or even conducting assessment on a mobile phone without the aid of an audiology expert (5, 8, 9).

Nonetheless, CA has been slow to gain clinical acceptance (2) despite the cost, access and time limitations with traditional pure-tone audiometry (PTA). Other limitations of diagnostic tests include instances where some patients have received normal hearing results yet experience hearing loss (10), and errors especially in bone conduction tests (11).

One of the other advantages that CA brings is the ability to augment existing PTA diagnostic tests with other additional factors in audiogram estimation such as altitude, tympanogram data, otoscopic images, gender, age, medication history, noise exposure and many others, to result in precision audiology at low cost, reduced time and at a scale that will meet the demands of society with increasing hearing loss challenges (1). For example, Cox and De Vries (12) used age and gender to speed the accuracy of audiogram estimation, and Zhao et al. (13) used noise exposure at work to improve the accuracy. However, there are few or no CA studies that have investigated how the different additional factors influence hearing loss between different population groups, nor how to transfer Machine Learning (ML) models from one population group to another without losing computational model performance.

This study focused on contributing to CA by introducing Transfer Learning (TL) to identify informative priors using audiogram databases from different population groups, and to investigate the extent to which the informative priors from the different population groups can speed up audiogram estimation. TL is an area of ML that focuses on utilizing knowledge gained while solving an ML problem and applying the same knowledge to a different but related problem. Through TL, new systems can quickly adapt to new situations, tasks and environments by re-using pre-trained models (14). Like the similar notion of transfer of learning in the education discipline, TL addresses how learning in one context can be applied to learning in another context, often with better or faster solutions. The key benefits of TL are the maximization of limited ML and data resources, the re-use of the ML and data resources for different tasks, the beneficial adaptation of ML models to a new environment or different context, and the ability to navigate beyond the growing constraints around data because of privacy laws. This means that through TL, audiogram estimation can be performed for significantly less cost and in less time.

This study sought to answer two main research questions. First, how can Transfer Learning (TL) be used to identify informative priors from different population groups? Second, how can the informative priors speed up audiogram estimation?

## 2 Data preparation

Table 1 describes the pure-tone audiogram databases that were used in the study. The data is proprietary and obtainable from the Scalable Hearing Rehabilitation for Low- and Middle-Income Countries (SHRLMIC), Project reference: UNOPS/CFP-2020/001/ATSCALE. The project was funded by the United States Agency for International Development (USAID) in support of the Global Partnership for Assistive Technology (ATscale) and managed by the United Nations Office for Project Services (UNOPS).

**Table 1:** Description of initial dataset of audiogram databases.

Database name	Audiograms	Brief description of database
Combined Age 1	2,571	Ages 18–40 combined audiograms
Combined Age 2	3,236	Ages 41–60 combined audiograms
Combined Age 3	4,796	Ages 61–80 combined audiograms
Combined Age 4	941	Ages >80 combined audiograms
Combined Income 1	400	Low income countries (only Malawi)
Combined Income 2	4,128	Lower middle-income countries (Cambodia, Egypt, India, Nepal, Philippines)
Combined Income 3	8,130	Upper middle-income countries (China, Dominican Republic, Indonesia, Jordan, Malaysia, Russia, Samoa, South Africa, Thailand and Turkey)
Combined Sensorineural Hearing Loss (SNHL)	8,261	Combined SNHL audiograms from the different populations, income groups and ages
Combined Total	11,544	Combined audiograms from the different populations, income groups and ages (excluding SNHL)
East Asia and Pacific with SHNL	2,493	Cambodia, China, Indonesia, Malaysia, Philippines, Samoa and Thailand with SHNL
East Asia and Pacific	3,490	Cambodia, China, Indonesia, Malaysia, Philippines, Samoa and Thailand
Europe and Central Asia with SHNL	2,588	Russia and Turkey with SHNL
Europe and Central Asia	3,392	Russia and Turkey
Latin America and the Caribbean with SNHL	517	Dominican Republic with SHNL
Latin America and the Caribbean	656	Dominican Republic
Middle East and North Africa with SNHL	672	Egypt and Jordan with SHNL
Middle East and North Africa	892	Egypt and Jordan
South Asia with SNHL	1,390	India and Nepal with SHNL
South Asia	2,318	India and Nepal
Sub-Saharan Africa with SNHL	608	Malawi and South Africa with SHNL
Sub-Saharan Africa	805	Malawi and South Africa

The project collected and aggregated audiogram data from various regions and countries, resulting in diverse and comprehensive audiogram databases. The data was also reorganized and classified collectively based on age and income groups, which allows for more nuanced analyses of global auditory health trends.

We decided to exclude the sensorineural hearing loss (SNHL) audiogram databases from the study due to their fundamental differences from normal conductive hearing loss audiograms. While the idea of transfer learning suggests that knowledge from one domain should be able to transfer to another, it was essential to consider the specific characteristics of each domain separately for now. SNHL and conductive hearing loss, while sharing knowledge and are related, exhibit distinct underlying physiological mechanisms and hearing profiles. By focusing on conductive hearing loss databases, we aimed to develop the model to the specific features and challenges of conductive hearing loss. We therefore considered that including SNHL audiograms in the model is an area for further research.

Duplicate audiograms and audiograms with missing values were removed. Outlier audiograms, that is, those with interaural gaps or ≥50 dB in two or more thresholds were purposefully left in the data similar to Parthasarathy et al. (15) and Charih et al. (16) in order to better represent the prior information in its original context. We then explored the statistical features (Table 2) using the six frequency octaves: 250 Hz, 500 Hz, 1 kHz, 2 kHz, 4 kHz and 8 kHz.

**Table 2:** Statistical characteristics of the audiogram databases.

Audiogram database	Statistic	Thresholds at frequencies ω=0.25,0.5,1,2,4,8 kHz
Combined Age 1	Interquartile range	[45 40 45 45 50 60]
	Median	[45 50 50 50 55 60]
	Mode	[25 25 60 120 120 120]
	Standard deviation	[28.0 29.4 31.2 33.1 33.9 35.6]
Combined Age 2	Interquartile range	[35 35 40 35 40 45]
	Median	[40 40 45 45 55 65]
	Mode	[20 25 25 30 60 120]
	Standard deviation	[25.8 27.5 27.4 27.5 28.6 31.0]
Combined Age 3	Interquartile range	[35 35 30 30 30 30]
	Median	[40 45 50 55 65 75]
	Mode	[25 45 50 55 65 120]
	Standard deviation	[24.4 24.2 24.1 23.6 23.3 25.1]
Combined Age 4	Interquartile range	[25 30 20 20 20 25]
	Median	[50 55 60 65 70 85]
	Mode	[45 50 60 65 70 120]
	Standard deviation	[21.3 20.4 18.9 17.6 18.3 20.4]
Combined Income 1	Interquartile range	[30 30 30 30 35 40]
	Median	[60 60 60 65 75 80]
	Mode	[55 55 55 65 120 120]
	Standard deviation	[25.5 26.9 25.8 26.6 27.1 27.6]
Combined Income 2	Interquartile range	[35 35 35 30 35 40]
	Median	[50 55 60 60 67.5 75]
	Mode	[40 50 60 60 70 120]
	Standard deviation	[25.1 25.5 26.0 26.6 27.3 29.7]
Combined Income 3	Interquartile range	[35 35 40 35 35 40]
	Median	[45 45 50 55 60 70]
	Mode	[25 50 55 60 60 120]
	Standard deviation	[24.78 25.9 26.3 26.7 27.2 29.9]
East Asia and Pacific	Interquartile range	[35 40 40 40 40 45]
	Median	[45 50 55 60 65 75]
	Mode	[20 25 60 65 75 120]
	Standard deviation	[27.04 27.0 27.4 27.9 28.6 31.1]
Europe and Central	Interquartile range	[30 30 30 30 35 35]
Asia	Median	[40 40 45 50 60 65]
	Mode	[25 40 50 50 60 65]
	Standard deviation	[22.8 24.4 23.7 24.0 25.2 27.6]
Latin America and the	Interquartile range	[32.5 40 40 40 50 55]
Caribbean	Median	[35 35 35 40 50 55]
	Mode	[25 25 25 25 25 25]
	Standard deviation	[24.64 26.5 29.1 30.0 29.9 32.3]
Middle East and North	Interquartile range	[35 35 40 40 35 45]
Africa	Median	[40 40 45 50 55 67.5]
	Mode	[25 35 25 25 40 120]
	Standard deviation	[24.1 25.0 26.7 28.0 28.2 30.6]
South Asia	Interquartile range	[35 30 35 30 35 40]
	Median	[50 55 55 60 65 75]
	Mode	[40 50 60 60 70 120]
	Standard deviation	[24.9 25.1 25.7 26.2 26.8 28.8]
Sub-Saharan Africa	Interquartile range	[40 40 30 30 35 35]
	Median	[45 50 55 55 65 75]
	Mode	[30 55 55 55 120 120]
	Standard deviation	[27.6 27.5 27.3 26.4 26.5 29.4]

## 3 Methods

We used an adaptive staircase method akin to the Hughson-Westlake (HW) method for its flexibility for use in computational modelling. The HW method uses reversals in intensity from increasing (or decreasing) intensities to identify the hearing thresholds. The more recent modified HW method is automated and remains popular among audiologists (17).

### 3.1 Transfer learning

There are three main tasks in TL. First, to identify “what” to transfer; second, to infer “how” to transfer; and third, to decide “when” to transfer based on whether TL was beneficial in the context.

#### 3.1.1 What to transfer

The starting point was to recognize that all the original and reclassified audiogram databases shared several features, particularly in using between 6–8 frequency octave intervals for both ears. This means that all audiogram databases have a degree of homogeneity. There is however also a degree of heterogeneity in the databases usually from the different additional features such as age, gender, location, noisy environment at work and others. While these associated features are not mandatory, they are valuable in ML methods to uniquely determine patterns such as audiogram phenotypes. The first step was therefore to identify the extent of homogeneity, whether marginal probability $P(X_{s}\cap X_{t})\neq 0$, and heterogeneity, whether $P(X_{s}\cap X_{t})=0$ across the different audiogram databases.

Table 2 reveals descriptive heterogeneity between the audiogram databases. It is particularly noticeable that the median threshold value occurs from 40 dB HL upwards across all the frequencies in all the databases. This finding supports the choice of using 40 dB HL as recommended by Maltby (18) as a preferable starting intensity and one that is not uncomfortable for all types of patients.

Despite the descriptive heterogeneity, it was necessary to use an inferential method to specifically determine the extent of homogeneity or heterogeneity. We opted for a Gaussian Mixture Model (GMM) to infer the phenotypes within each of the databases because GMMs allow for soft clustering, that is, a data point can belong to more than one cluster in degrees (19). This is different from hard clustering where a data point belongs to strictly one cluster. GMMs are therefore ideal for modeling audiogram phenotype categories. Previous work by Cox and De Vries (12) used a GMM to introduce an informative prior as a prior distribution p(t|a,g) in audiogram assessment conditioned on age a∈N and gender g∈{female|male}.

GMMs lean more towards being probabilistic distributions rather than being models because they are a combination of different Gaussian distributions (20). GMMs model discrete latent variables as a linear combination of Gaussians (20) in the form:vv

(1) $$p\text{( x) }=\sum_{k=1}^{K}\pi_{k}N\left(x|\mu_{k},\Sigma_{k}\right)$$

where the Gaussian density $N(x|\mu_{k},\Sigma_{k})$ is a component of the mixture and has its own mean $\mu_{k}$ and covariance $\Sigma_{k}$. $\pi_{k}$ is the mixing coefficient that represents the degree to which a data point fits within different clusters and is a valid probability such that $\sum_{k=1}^{K}\pi_{k}=1\text{ \textbackslash{};and\textbackslash{}; }0\leq \pi_{k}\leq 1$.

The GMM is therefore governed by the parameters π,μ \;and\; Σ. The setting of the parameters using maximum likelihood makes the solution analytically complex (20) and takes it out of the closed form. Thus, our goal becomes the minimization of the negative log-likelihood loss function, L 2:

(2) $$L(\pi ,\mu ,\Sigma )=-\sum_{i=1}^{n}\log\left(\sum_{k=1}^{K}\pi_{k}N(x_{i}|\mu_{k},\Sigma_{k})\right)+\lambda \sum_{k=1}^{K}\|\Sigma_{k}{\|}^{2}$$

where λ is a regularization parameter. We included a regularization value of 0.01 in the loss function to ensure that the covariance matrix remained positive semi-definite.

We therefore used the Expectation Maximization (EM) method because of its ability to estimate the parameters in an iterative and efficient way.

**Figure 1:**
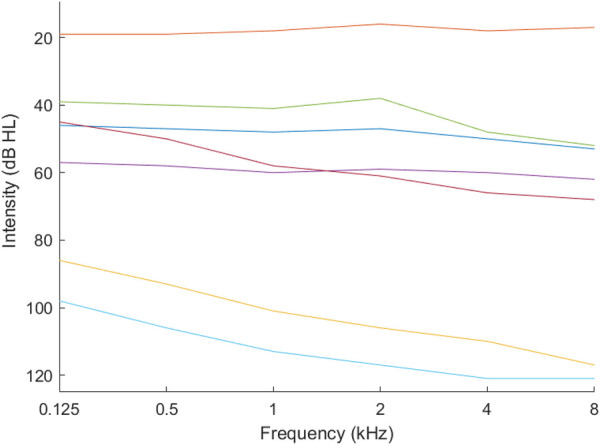
Phenotypes for ages 18–40 years.

**Figure 2:**
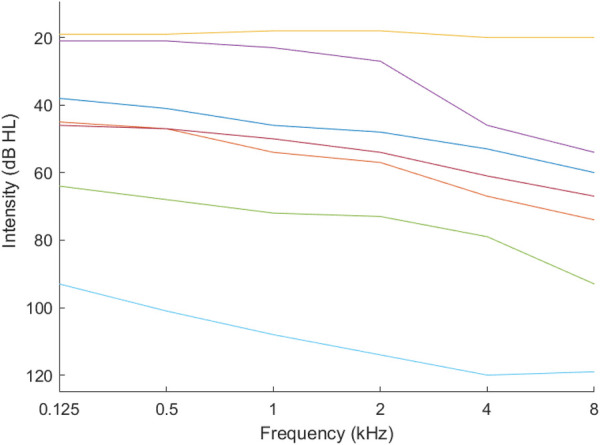
Phenotypes for combined income — upper middle income countries.

We used k-means clustering with the *gap* statistic (21) to identify the optimum number of K phenotypes. Figures 1, 2 present example phenotypes using the number of clusters identified using the k-means clustering method, in these examples, both 7 clusters. The two figures visually reveal the heterogeneity. Table 3 presents the phenotypes using the number of clusters identified and the $\pi_{k}$ as the mixing coefficients of each cluster.

**Table 3:** Phenotypes of the audiogram databases and their characteristics.

Audiogram database	Phenotype features	Number of clusters and corresponding mixing coefficients in []
Combined Age 1	Number of clusters, k	7
	$\pi_{k}$, the mixing coefficients	[0.1745 0.1365 0.0498 0.2489 0.1255 0.0847 0.1802]
Combined Age 2	Number of clusters, k	7
	$\pi_{k}$, the mixing coefficients	[0.1881 0.1349 0.1356 0.3039 0.0495 0.0325 0.1556]
Combined Age 3	Number of clusters, k	7
	$\pi_{k}$, the mixing coefficients	[0.0840 0.0927 0.0856 0.3458 0.1563 0.1007 0.1349]
Combined Age 4	Number of clusters, k	6
	$\pi_{k}$, the mixing coefficients	[0.0963 0.0669 0.1726 0.4903 0.1595 0.0144]
Combined Income 1	Number of clusters, k	6
	$\pi_{k}$, the mixing coefficients	[0.1179 0.1881 0.3561 0.0351 0.1200 0.1827]
Combined Income 2	Number of clusters, k	7
	$\pi_{k}$, the mixing coefficients	[0.1632 0.1137 0.0048 0.1054 0.2005 0.1192 0.2932]
Combined Income 3	Number of clusters, k	7
	$\pi_{k}$, the mixing coefficients	[0.1216 0.1348 0.0611 0.1085 0.2033 0.0476 0.3231]
East Asia and Pacific	Number of cluster, k	7
	$\pi_{k}$, the mixing coefficients	[0.1137 0.3674 0.1465 0.0297 0.1109 0.1531 0.0787]
Europe and Central	Number of clusters, k	7
Asia	$\pi_{k}$, the mixing coefficients	[0.2128 0.0856 0.1047 0.0676 0.2176 0.2125 0.0992]
Latin America and the	Number of clusters, k	6
Caribbean	$\pi_{k}$, the mixing coefficients	[0.0911 0.0290 0.1014 0.2984 0.1821 0.2980]
Middle East and North	Number of clusters, k	4
Africa	$\pi_{k}$, the mixing coefficients	[0.2238 0.1132 0.6084 0.0546]
South Asia	Number of clusters, k	7
	$\pi_{k}$, the mixing coefficients	[0.1777 0.0302 0.1514 0.3274 0.0275 0.1443 0.1416]
Sub-Saharan Africa	Number of clusters, k	7
	$\pi_{k}$, the mixing coefficients	[0.0919 0.1473 0.2313 0.0099 0.0993 0.2410 0.1793]

Based on the number of clusters and the differences in the mixing coefficients (Table 3), and therefore the phenotypes, the audiogram databases were confirmed as heterogeneous, that is, the marginal probabilities $P(X_{s}\cap X_{t})=0$. The covariance matrix sets were also heterogeneous, and the mean values in the component models were all different which also pointed to heterogeneity.

This finding limited the TL approach to feature-based options. The task was therefore to learn the mapping function $f_{s}(\cdot )\to f_{t}(\cdot )$ between the audiogram databases and the audiogram estimation required.

#### 3.1.2 How to transfer

The results of the preceding step, “what to transfer”, are used to inform the “how to transfer”, whether through common features, instances, models or parameters.

We therefore turned to active learning, particularly stream-based active learning to identify informative features from the audiogram databases. In active learning, there is an intentionality about the profile being searched for in every query; that is, the next query is based on answers to the previous query (22). Based on the sets of answers, the model or classifier is improved continually.

**Figure 3:**
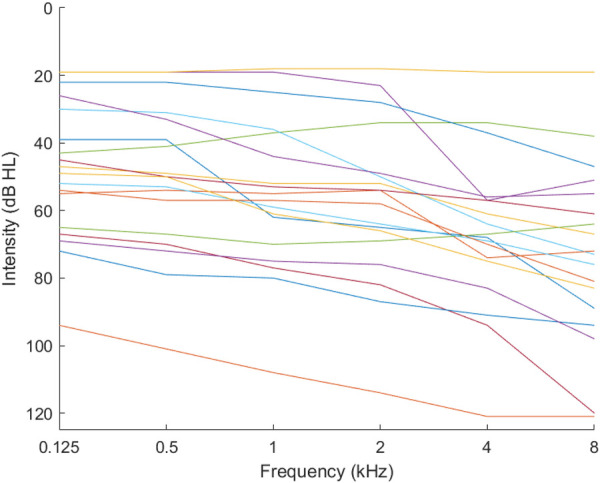
Phenotypes for combined audiograms.

We combined all the audiogram databases into one large dataset, and used the large dataset as the source data, $D_{s}$, from which to discover and extract good features to use in the audiogram estimation. Using the GMM, we discovered 18 phenotypes (Figure 3).

We regarded the profile being searched for as that which is generated from the feature-based TL mapping function aimed at learning a pair of mapping functions $\left\{\;f_{s}(\cdot ),f_{t}(\cdot )\right\}$ to map data respectively from $D_{s}$ and $D_{t}$ to a common feature space, $D_{c}$, ($f_{s}(D_{s})\mapsto D_{c}$ and $f_{s}(D_{t})\mapsto D_{c}$) where the difference between $D_{s}$ and $D_{t}$ can be reduced. $D_{c}$ then serves as the input to the ML algorithm. We used feature augmentation (14, 23) with set membership where the common feature space is augmented at every new query:

(3) $$D_{t}\subset D_{c}=\frac{1}{n}\sum_{i=i}^{n}\left[\{(x_{t_{i}}+\varepsilon {)}_{i=1}^{n_{t}}\}\in D_{s}\right]$$

where n are the number of features, and ε is the error term which in our study is an informativeness measure.

The combination, or active transfer learning (ATL), allows for features identified using TL to be accepted or discarded by the active learner based on the informativeness measure. We adopted the informativeness measure ε as a range of 5% between neighbouring frequencies. This is because thresholds for each frequency in normal hearing or conductive hearing loss are usually close to each other (24). This phenomenon, where thresholds for each frequency are typically closely grouped together, is referred to as unilateral conductive hearing loss (24). The low informativeness measure means that once a frequency threshold is discovered, the neighbouring frequencies can be estimated using the same informativeness measure. The next section outlines the ATL algorithm applied to an adaptive staircase method akin to the HW method.

#### 3.1.3 Active transfer learning with the adaptive staircase method akin to the Hughson-Westlake method

**Figure 4:**
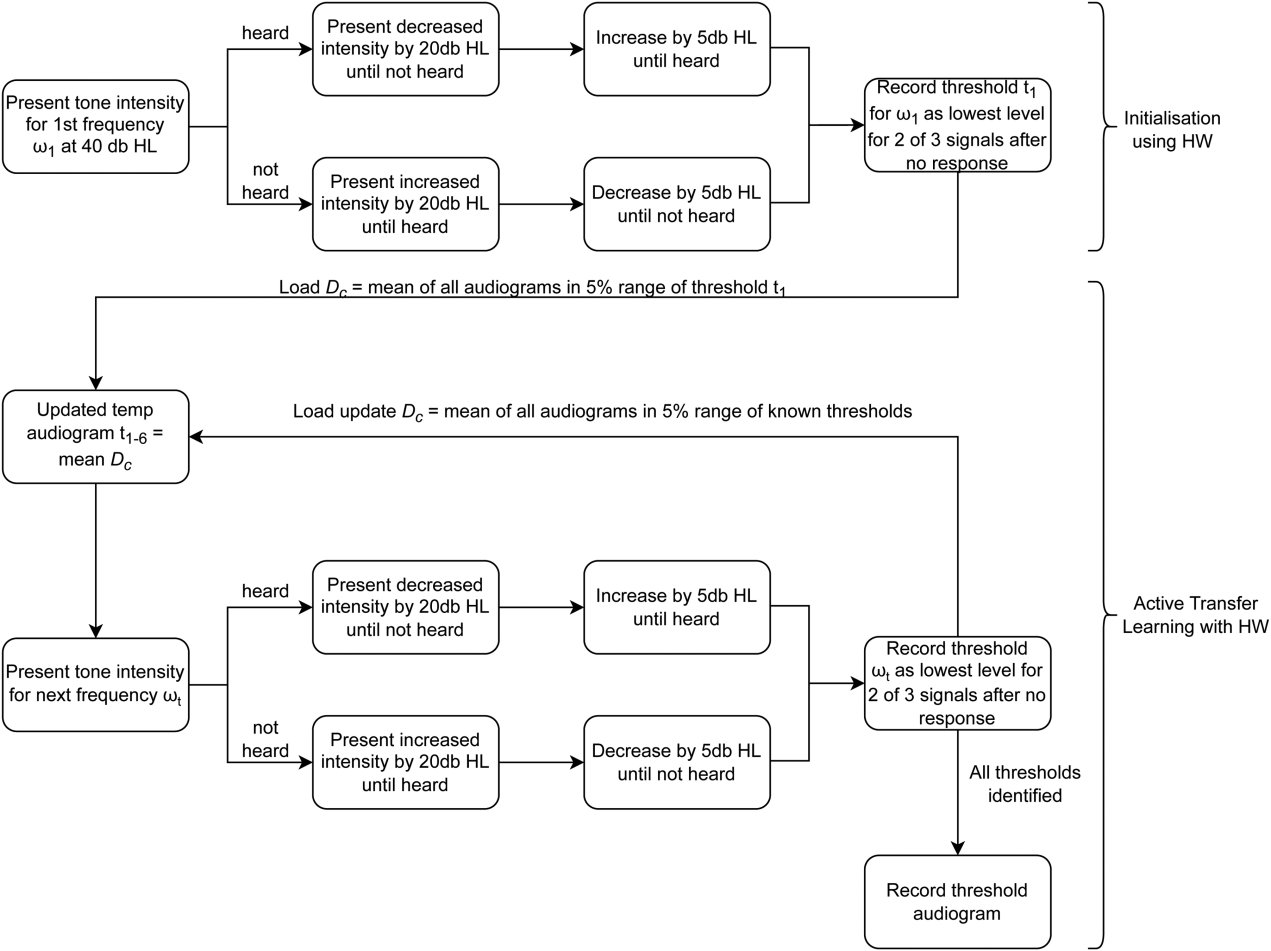
Algorithm for ATL with HW.

First, the starting frequency is initialized at 40 dB HL, then decreased (or increased) in steps of 20 dB HL until there is a reversal (heard/not heard). For initialisation, the adaptive staircase procedure is followed until the threshold for the starting frequency $t_{1}$ is identified. $t_{1}$ is used to identify all audiograms from $D_{s}$ within 5% of $t_{1}$, which then becomes K. The mean of K then offers the starting intensity for the proceeding frequencies. The process is repeated until all the thresholds are identified. Figure 4 summarizes the ATL algorithm.

## 4 Results

We used a Dell Laptop computer with an Intel(R) Core(TM) i5-8265U CPU 1.6GHz 1.80GHz. The memory capacity was 8GB. It had a 64-bit Operating System with an x64-based processor running Windows 10 version 2004 (OS Build 19041.1415). The Hard Disk was a 473GB hard drive with 72.2GB of free space.

### 4.1 Large interaural gaps

The data in the Europe and Central Asia audiogram database had several audiograms with large interaural ranges exceeding 50 dB. Specifically, 926 audiograms had interaural gaps of ≥50 dB, with three audiograms having gaps of 110 dB. In this study, these interaural gaps were allowed. In later studies, a decision can be made to eliminate these gaps as they tend to distort the phenotypes.

### 4.2 Cleaning up the data

The same database had several incorrect audiogram entries, with one recorded at 510 dB for the 500 Hz frequency and many others recorded strangely as 6, 8, 10, 11 and 12, which we infer could have been incorrect entries for 60, 80, 100, 110 and 120. Other strange values included 66, 81 and 21. These errors seemed as though they were manually inserted, hence introducing human error. These data anomalies were manually deleted as part of the data preparation stage.

The anomalies reveal the importance of preparing data and creating a database with verified and cleaned audiograms. This is important because stream-based active learning is sensitive to such errors.

### 4.3 Increased phenotypes

We also found that the number of clusters (k ) that could be identified using the k-means clustering method could be increased to 18 clusters, as seen in Figure 3. Nonetheless, we limited the number of clusters to 7 for each database to remain consistent with the WHO standard of seven phenotypes (25) and allowed the combined database to identify 18 clusters (phenotypes).

However, we note that as ML becomes more accessible, the concept of phenotypes might need to be considered as precision audiometry will allow multiple and various types of classifications which might not lend themselves to carefully curated phenotypical classes.

### 4.4 Accuracy and reliability benchmarks

We present the results of similar studies against which we benchmarked this study. Song et al. (26) performed similar audiogram estimation simulations using Halton samples (low discrepancy sequences that appear random). We use the results from that study to benchmark our simulation results. Tables 4, 5 present the accuracy and reliability results that are used to benchmark our results.

**Table 4:** Accuracy and reliability for 200 Halton samples in Song (26).

Frequency (kHz)	0.25	0.5	1	2	4	8
Mean α absolute error (dB)	1.53	1.60	1.70	1.86	2.47	3.32
Std α absolute error (dB)	1.03	1.09	1.17	1.32	1.90	2.60

**Table 5:** Accuracy and reliability of different Halton sample sizes in Song (26).

Number of samples	20	50	100	200	500	1,000
Mean α absolute error (dB)	6.63	4.35	3.03	2.08	1.30	0.933
Std α absolute error (dB)	5.42	7.28	2.57	1.74	1.23	0.913

Song et al.’s (3) experiments with 21 participants gave 78.4 stimuli presentations ±11 dB for both ears. Table 6 presents their results.

**Table 6:** Accuracy and reliability for 21 participants in Song (26).

Frequency (kHz)	0.25	0.5	1	2	4	8	All
Mean differences and standard deviations							
Mean difference (dB HL)	−0.15	1.55	1.63	0.26	1.03	0.03	0.75
Standard deviation (dB HL)	6.27	7.03	4.14	5.34	6.78	8.11	6.29
Average absolute differences and deviations							
Mean absolute difference (dB HL)	4.80	5.05	3.58	3.95	3.95	5.03	0.75
Standard deviation (dB HL)	3.97	5.07	2.60	3.55	4.59	6.52	4.45
Median absolute difference (dB HL)	5.00	3.00	3.00	3.00	4.00	3.00	3.00
Interquartile range (dB HL)	4.00	6.00	3.50	4.00	4.00	5.00	4.00

Barbour et al.’s (27) experiments on 21 participants using the same methods of Song et al. (3) implemented in an online platform yielded a mean absolute difference between thresholds of 3.2 presentations ±5.15 dB. Heisey et al. (28) use a modified version of Song et al. (3) for a mean absolute difference between masked and unmasked experiments of under 5 dB at all frequencies with an overall mean of 3.4 presentations ±2.7 dB.

### 4.5 Simulations of HW and ATL

We performed ATL on randomly selected sets of 2, 5, 20, 56 and 556 audiograms from each of the 18 phenotypes identified in the combined audiogram database. This gave 36, 90, 360, 1,008 and 10,008 simulations, respectively. Table 7 shows the mean, standard deviation, minimum, maximum and mode number of stimuli presentations for the simulations of 36 audiograms. Table 8 shows the results for the counts, and Tables 9–13 shows the accuracy and reliability results for the different simulations.

**Table 7:** Stimuli presentations for the simulation of 36 audiograms (15 examples are shown).

Audiogram	HW	ATL
[5 10 5 0 10 10]	58	23
[25 35 30 30 25 20]	33	25
[20 30 30 25 30 25]	34	18
[25 25 25 20 25 25]	37	18
[10 10 10 5 15 20]	52	21
[15 15 15 15 20 15]	47	17
[30 30 25 30 15 30]	34	21
[45 40 25 25 25 35]	33	28
[15 30 40 40 35 45]	34	25
[25 25 30 20 15 10]	41	26
[35 35 30 40 35 40]	29	22
[40 20 20 20 15 10]	44	40
[25 20 25 20 25 25]	38	18
[25 25 30 20 15 10]	41	25
[15 30 40 45 45 50]	36	27
Mean presentations	41.3	22.0
Standard deviation (dB)	9.02	4.75
Minimum presentations	28	15
Maximum presentations	66	40
Mode presentations	34	18

**Table 8:** Stimuli presentations for the different audiogram simulations.

Simulations	36	90	360	1,008	10,008
Mean presentations	25.3	25.2	25.3	25.2	25.3
Standard deviation (dB)	1.06	1.06	1.06	1.08	1.07
Minimum presentations	24	24	24	24	24
Maximum presentations	28	28	29	30	35
Mode presentations	25	25	25	25	25

**Table 9:** Accuracy and reliability for 36 simulations.

Frequency (kHz)	0.25	0.5	1	2	4	8	Mean	Mean (abs)
Mean (dB)	0.11	0.14	0.39	0.22	0.19	0.00	0.18	1.24
Minimum (dB)	−3.00	−2.00	−3.00	−2.00	−3.00	−3.00	−1.50	0.50
Maximum (dB)	3.00	3.00	3.00	2.00	4.00	3.00	1.67	2.00
Mode (dB)	1.00	−1.00	1.00	1.00	0.00	1.00	0.17	1.17
Standard deviation (dB)	1.69	1.57	1.46	1.31	1.49	1.57	0.64	0.36
Interquartile range (dB)	2.50	2.00	2.50	2.00	1.50	2.00	0.83	0.58

**Table 10:** Accuracy and reliability for 90 simulations.

Frequency (kHz)	0.25	0.5	1	2	4	8	Mean	Mean (abs)
Mean (dB)	0.29	0.47	0.20	0.21	0.59	−0.03	0.29	1.29
Minimum (dB)	−3.00	−2.00	−3.00	−3.00	−3.00	−3.00	−1.00	0.67
Maximum (dB)	3.00	3.00	2.00	3.00	4.00	3.00	1.33	2.17
Mode (dB)	−1.00	1.00	−1.00	1.00	2.00	1.00	−0.17	1.33
Standard deviation (dB)	1.53	1.59	1.45	1.37	1.68	1.56	0.56	0.33
Interquartile range (dB)	3.00	3.00	2.00	2.00	3.00	2.00	1.00	0.50

**Table 11:** Accuracy and reliability for 360 simulations.

Frequency (kHz)	0.25	0.5	1	2	4	8	Mean	Mean (abs)
Mean (dB)	0.49	0.43	0.15	0.15	0.39	0.19	0.30	1.34
Minimum (dB)	−3.00	−2.00	−3.00	−3.00	−5.00	−3.00	−1.33	0.50
Maximum (dB)	4.00	3.00	3.00	4.00	4.00	3.00	1.83	2.67
Mode (dB)	2.00	1.00	0.00	0.00	−1.00	1.00	0.50	1.17
Standard deviation (dB)	1.64	1.59	1.47	1.52	1.75	1.65	0.62	0.39
Interquartile range (dB)	3.00	3.00	2.00	2.00	3.00	2.00	0.83	0.58

**Table 12:** Accuracy and reliability for 1,008 simulations.

Frequency (kHz)	0.25	0.5	1	2	4	8	Mean	Mean (abs)
Mean (dB)	0.39	0.45	0.27	0.09	0.22	−0.09	0.22	1.32
Minimum (dB)	−3.00	−2.00	−3.00	−3.00	−5.00	−125.00	−19.50	0.17
Maximum (dB)	4.00	3.00	3.00	4.00	4.00	4.00	2.00	22.17
Mode (dB)	1.00	1.00	0.00	0.00	−1.00	0.00	0.33	1.33
Standard deviation (dB)	1.65	1.54	1.36	1.57	1.71	4.24	0.87	0.75
Interquartile range (dB)	3.00	3.00	2.00	2.00	3.00	2.00	0.83	0.50

**Table 13:** Accuracy and reliability for 10,008 simulations.

Frequency (kHz)	0.25	0.5	1	2	4	8	Mean	Mean (abs)
Mean (dB)	0.35	0.45	0.07	0.11	0.27	−0.01	0.21	1.37
Minimum (dB)	−3.00	−2.00	−125.00	−125.00	−10.00	−125.00	−43.50	0.17
Maximum (dB)	4.00	3.00	4.00	4.00	4.00	4.00	2.33	44.17
Mode (dB)	1.00	1.00	0.00	0.00	−1.00	0.00	0.33	1.33
Standard deviation (dB)	1.64	1.54	4.73	2.37	1.72	4.62	1.32	1.23
Interquartile range (dB)	3.00	3.00	2.00	2.00	3.00	2.00	0.83	0.50

ATL had lower stimuli presentation variability compared with HW (Table 7) as seen in the improved lower mean of 22.0 presentations with a reliability using standard deviation of ±4.75 dB. The result is also lower than Song et al. (3) with an average of 78.4 presentations ±11 dB for both ears, which, when halved, is 39.2 presentations. ATL also had a lower minimum stimuli presentation of 15 compared with the HW minimum count of 28 (Table 7), while for the different simulations between 90 to 10,008, ATL had a minimum stimuli presentation of 25.2±1.06 dB and a maximum of 25.3±1.06 dB.

The ATL accuracy was evaluated using the non-parametric numerical 50% probability point from the mean difference and mean absolute difference (Tables 9–13). Reliability was measured using the 25–75% interquartile range (29). The results reveal that ATL accurately determined the threshold at each frequency with very small margins of error of less than 5 dB. ATL was also consistent with much less volatility in its results across all the frequencies also giving a spread of less than 5 dB as well. Even with the simulation of 10,008 audiograms where some of the minimums were more than 125 dB, the spread was still less than 5 dB.

## 5 Discussion and conclusions

We investigated the influence of informative priors derived from audiogram databases of diverse populations, with an emphasis placed on understanding the degree to which these informative priors can improve CA models without losing computational performance. Specifically, we hypothesized that TL could offer informative priors that improve the accuracy of probabilistic CA models.

The key finding was that Transfer Learning offers an appropriate means to combine audiogram databases to extract meaningful informative priors that can be used in CA. The finding answers Wasmann et al.’s (2) suggestion to find ways to maximise the disparate audiogram databases from around the world.

We offer ATL as a reliable and consistent approach to leverage audiograms from multiple databases to speed up audiogram estimation, in this study improving HW tests from a mean 41.3 presentations ±9.02 dB down to 25.3 presentations ±1.04 dB. The reliability and time improvements were also better compared to other existing CA models.

We also found that different population groups have different phenotypes, and therefore are unlikely to share computational parameters or hyperparameters. This means that CA methods on audiogram estimation are likely to become more data-driven compared with being model-driven.

ML methods also produced a varying number of phenotypes from each population according to the data in the dataset. Further research with other ML methods should look into this.

This use of TL to speed audiogram estimation is heavily dependent on the cleanliness of the data. It reveals the importance of preparing data and the necessity to create a database with verified clean audiograms. One of the ways of achieving this is through automating data capture of audiogram records.

We defined an approach as to *when to transfer* in TL; which is one of the primary problems in TL (14). We also identified *what to transfer*; the intensity of adjacent frequencies using an exploration mechanism derived from active learning. We then identified the *how to transfer* using an algorithm which uses any identified intensity in one frequency to predict the intensity in the adjacent frequencies.

### 5.1 Limitations

We did not cater for imbalanced datasets and hence greater accuracy can be achieved by incorporating algorithms that take into account the imbalances in the datasets. For example, data from Malawi was limited, that is, it had only 400 audiograms.

### 5.2 Areas for further research

The k number of clusters was intentionally limited to seven for the individual databases in accordance with the WHO recommended number of phenotypes (25). However, the GMM cluster evaluation using k-means clustering yielded up to 18 clusters from the combined audiogram database. This shows, similar to Parthasarathy et al. (15) who identified 10 clusters in their data, that further research can be attempted with a higher number of phenotypes for higher precision. This also brings into question the notion of human audiogram phenotypes considering that ML methods are only getting more advanced. We also found that ATL is sensitive to negative thresholds and tends to overshoot. This phenomenon needs to be investigated further. We also propose that ATL could be extended to other population databases, to include SNHL audiograms, and to include additional information such as altitude and noise exposure.

## Data Availability

The original contributions presented in the study are included in the article/Supplementary Material, further inquiries can be directed to the corresponding author.
